# Challenges and implications of the use of artificial intelligence in health care, with an emphasis on nursing. Scoping review

**DOI:** 10.17533/udea.iee.v43n3e15

**Published:** 2025-11-06

**Authors:** Neelam Shah, Jyoti Bala, Ashutosh Sharma, Jitendra Singh Parmar, Dharmendra Singh

**Affiliations:** 1 Faculty of Nursing, Email: shahneelam196@gmail.com https://orcid.org/0009-0001-8785-1785 Uttar Pradesh University of Medical Sciences Faculty of Nursing India shahneelam196@gmail.com; 2 Faculty of Nursing, Email: jb1849@gmail.com https://orcid.org/0000-0003-1732-0636 Uttar Pradesh University of Medical Sciences Faculty of Nursing India jb1849@gmail.com; 3 Faculty of Nursing, Email: ashutosh_sharma2008@rediffmail.com. https://orcid.org/0009-0000-8543-4921 Uttar Pradesh University of Medical Sciences Faculty of Nursing India ashutosh_sharma2008@rediffmail.com; 4 Faculty of Nursing, Email: singhjitu222@gmail.com https://orcid.org/0009-0002-4868-6438 Uttar Pradesh University of Medical Sciences Faculty of Nursing India singhjitu222@gmail.com; 5 Faculty of Nursing, Email: dharmendrasingh8023@gmail.com. https://orcid.org/0009-0003-2652-5358 Uttar Pradesh University of Medical Sciences Faculty of Nursing India dharmendrasingh8023@gmail.com; 6 Uttar Pradesh University of Medical Sciences, Saifai, Uttar Pradesh, India. Uttar Pradesh University of Medical Sciences Uttar Pradesh University of Medical Sciences Saifai Uttar Pradesh India

**Keywords:** maternal health, prenatal care, maternal health services, maternal mortality, nursing care., salud materna, atención prenatal, servicios de salud materna, mortalidad materna, atención de enfermería., saúde materna, cuidados pré-natais, serviços de saúde materna, mortalidade materna, cuidados de enfermagem.

## Abstract

**Objective.:**

To review the literature related to ethics of artificial intelligence (AI) in healthcare, with a particular emphasis on its challenges and implications in nursing.

**Methods.:**

Data bases including PubMed, Scopus, Web of Science, and CINAHL are reviewed. Inclusion criteria focused on English-language articles addressing AI ethics in healthcare, with priority given to empirical studies, World Health Organization (WHO) reports, and nursing-specific scholarship. General Search Items included artificial intelligence ethics, AI in healthcare challenges, nursing AI implications, algorithmic bias healthcare, informed consent AI, privacy data protection AI, and WHO AI guidelines, combined with Boolean operators (e.g., "AI AND nursing autonomy") and filters for publication date (post-2018) and article type (reviews, originals).

**Results.:**

Most of the studies emphasizes that integration of Artificial intelligence provides substantial benefits for patients, medical professionals, and the overall healthcare framework. Like the improving the primary healthcare, cost reduction, and enhanced efficiency of medical and clinical processes and it also helps where human intelligence is needed i.e. analytical reasoning, acquiring knowledge, and decision-making. While it offers immense possibilities, this technology demands vast amounts of patient information, leading to concerns about confidentiality, protection, and other moral dilemmas. It also highlights the need for nurses to develop AI literacy and bias recognition to balance technological efficiency with humanistic care and ethical evaluation; enabling nurses to monitor unethical AI applications and ensure fairness in patient care.

**Conclusion.:**

AI is revolutionizing the healthcare sector but demands robust ethical governance to mitigate harms like discrimination and privacy erosion. For nursing, proactive integration-via updated curricula and interdisciplinary policies-can foster safe, equitable AI adoption, ultimately advancing human dignity and health outcomes.

## Resumen

**Objetivo.:**

Revisar la literatura relacionada con la ética de la inteligencia artificial (IA) en la atención sanitaria, con especial énfasis en sus retos e implicaciones en la enfermería.

**Métodos.:**

Se revisaron las bases de datos como PubMed, Scopus, Web of Science y CINAHL. Los criterios de inclusión se centraron en artículos en inglés que abordaban como tema la ética de la IA en la atención sanitaria, dando prioridad a los estudios empíricos, los informes de la Organización Mundial de la Salud (OMS) y los trabajos académicos específicos sobre enfermería. Los términos de búsqueda generales incluyeron ética de la inteligencia artificial, retos de la IA en la atención sanitaria, implicaciones de la IA en la enfermería, sesgo algorítmico en la atención sanitaria, consentimiento informado en la IA, protección de datos y privacidad en la IA y directrices de la OMS sobre la IA, combinados con operadores booleanos (por ejemplo, «IA Y autonomía de la enfermería»), filtros por fecha de publicación (posterior a 2018) y por tipo de artículo (revisiones, originales).

**Resultados.:**

La mayoría de los estudios señalan que la integración de la IA proporciona beneficios sustanciales para los pacientes, los profesionales de la salud y el marco sanitario en general. Por ejemplo, mejora la atención primaria, reduce los costos y aumenta la eficiencia de los procesos clínicos, y también ayuda en aquellos ámbitos en los que se necesita la inteligencia humana, como el razonamiento analítico, la adquisición de conocimientos y la toma de decisiones. Aunque ofrece inmensas posibilidades, esta tecnología exige grandes cantidades de información sobre los pacientes, lo que suscita preocupaciones sobre la confidencialidad, la protección y otros dilemas morales. También se destaca la necesidad de que las enfermeras desarrollen la alfabetización en IA y el reconocimiento de sesgos con el fin de equilibrar la eficiencia tecnológica con el cuidado humanizado. La evaluación ética permitirá a las enfermeras monitorizar las aplicaciones poco éticas de la IA y garantizar la equidad en la atención al paciente.

**Conclusión.:**

El uso de la IA está revolucionando el sector sanitario, y exige una sólida gobernanza ética para mitigar los posibles daños de su empleo. En el caso de la enfermería, la integración proactiva, mediante la actualización de los planes de estudio, puede fomentar la adopción segura y equitativa de la IA, lo que en última instancia promoverá la dignidad humana y los resultados en materia de salud.

## Resumo

**Objetivo.:**

Revisar a literatura relacionada à ética da inteligência artificial (IA) na saúde, com ênfase particular em seus desafios e implicações para a enfermagem.

**Métodos.:**

Foram revisadas bases de dados como PubMed, Scopus, Web of Science e CINAHL. Os critérios de inclusão se concentraram em artigos em inglês que abordassem a ética da IA ​​na saúde, priorizando estudos empíricos, relatórios da Organização Mundial da Saúde (OMS) e trabalhos acadêmicos específicos da enfermagem. Os termos gerais de busca incluíram ética da inteligência artificial, desafios da IA ​​na saúde, implicações da IA ​​para a enfermagem, viés algorítmico na saúde, consentimento informado em IA, proteção de dados e privacidade em IA e diretrizes da OMS sobre IA, combinados com operadores booleanos (por exemplo, "IA E autonomia da enfermagem"), filtros por data de publicação (após 2018) e tipo de artigo (revisões, originais).

**Resultados.:**

A maioria dos estudos indica que a integração da IA ​​oferece benefícios substanciais para pacientes, profissionais de saúde e para o sistema geral de saúde. Por exemplo, melhora a atenção primária, reduz custos e aumenta a eficiência dos processos clínicos, além de auxiliar em áreas onde a inteligência humana é necessária, como raciocínio analítico, aquisição de conhecimento e tomada de decisão. Embora ofereça imensas possibilidades, essa tecnologia requer grandes quantidades de informações do paciente, levantando preocupações sobre confidencialidade, proteção e outros dilemas morais. A necessidade de enfermeiros desenvolverem alfabetização em IA e reconhecimento de vieses também é destacada para equilibrar a eficiência tecnológica com o cuidado humanizado. A avaliação ética permitirá que enfermeiros monitorem aplicações antiéticas de IA e garantam a equidade no atendimento ao paciente.

**Conclusão.:**

O uso de IA está revolucionando o setor de saúde e requer forte governança ética para mitigar os danos potenciais de seu uso. Na enfermagem, a integração proativa por meio de atualizações curriculares pode promover a adoção segura e equitativa da IA promovendo, em última análise, a dignidade humana e os resultados de saúde.

## Introduction

Artificial intelligence (AI) is the replication of simulation of human intelligence, behavior, instant computations, troubleshooting in machines based on available data.^1^ Artificial intelligence in healthcare have primarily transmuted the medical domain, encompassing the diagnostics, Maintenance of digital records, treatment, enhancing health professionals' intelligence, novel drug discovery, comprehensive biological data analysis, accelerating the workflow, facilitating data storage and retrieval for healthcare institutions. AI driven innovation possesses the potential to shape the future of industries and humanity, but it is a double-edged blade.^2^ AI is quickly being adopted in the healthcare, bringing forth a revolutionary phase of innovation that is set to transform how we diagnose, cure, and manage ailments.^3^ AI-driven technology provides substantial benefits for patients, medical professionals, and the overall healthcare framework. Like the improving the primary healthcare, cost reduction, and enhanced efficiency of medical and clinical processes and it also helps where human intelligence is needed i.e. analytical reasoning, acquiring knowledge, and decision-making.^4^ Besides this, artificial intelligence encounters numerous ethical and legal problems, also it is inaccessible to all populations specially the low-income and developing nations still.^5^^,^^6^ It is essential to acknowledge that ethical conflicts, privacy concerns, data security, informed consent, societal disparities, medical counseling, compassion, and empathy are some of the challenges associated with adoption of artificial intelligence.^7^^,^^8^ Thus, before incorporating artificial intelligence into the healthcare , health professionals and experts need to adhere to the four fundamental principles of ethics i.e. autonomy, beneficence, nonmaleficence, and justice-across all planes of healthcare.^9^

The World Health Organization (WHO) has released two reports, in 2021 and 2023, on AI applications in healthcare. These reports outline key principles and considerations for the responsible and ethical use of AI. It is essential that AI in healthcare is designed and utilized in a manner that upholds human dignity, core rights, and values. AI systems should promote fairness, inclusivity, transparency, and accountability. Additionally, the WHO’s findings highlight existing legal and ethical gaps in AI regulation within the healthcare sector. The use of AI in medicine presents issues of trust, accountability, discrimination risks, privacy concerns, and patient autonomy, alongside its many potential advantages and disadvantages.^3^ AI is an innovative technology with the potential to transform healthcare services. However, its benefits will only be fully realized if ethical oversight is incorporated into its design, development, and application in clinical settings.^4^

## Methods

Data bases including PubMed, Scopus, Web of Science, and CINAHL are reviewed. Inclusion criteria focused on English-language articles addressing AI ethics in healthcare, with priority given to empirical studies, World Health Organization (WHO) reports, and nursing-specific scholarship. General Search Items included artificial intelligence ethics, AI in healthcare challenges, nursing AI implications, algorithmic bias healthcare, informed consent AI, privacy data protection AI, and WHO AI guidelines, combined with Boolean operators (e.g., "AI AND nursing autonomy") and filters for publication date (post-2018) and article type (reviews, originals).

## Results

### Prerequisite of artificial intelligence in healthcare

Artificial intelligence has the capability to analyze huge amounts of digital data at a speed and efficiency beyond human capacity, Hence Artificial intelligence has the potential to transform the healthcare sector by uncovering valuable and essential insights and help health professionals to concentrate on actual patient concerns while delegating tasks that can be automated to computer systems. There are some examples of uses of Artificial intelligence (AI) in various areas of healthcare are:^5^**(i) Diagnosis:** With the help of Imaging technology, AI can help to detect lung cancer, pneumonia, and diabetic retinopathy in CT scans, chest X-rays, and retinal scans, AI can help in diagnosing cancer from tissue samples Surgery; **(ii) Robot-assisted surgery:**AI-controlled robots can perform complex operations with greater precision and dexterity; **(iii) Documentation:** AI can quickly analyze patient information and suggest required improvements to be done in documentation, saving time and reducing errors; **(iv) Drug discovery:** AI can speed up drug discovery and diagnoses personalized medicine; and **(v) Virtual nursing assistants:** AI can also interact with patients, answer their queries, and help health professionals to understand the patient conditions_._

### Ethical Principles related to use of Artificial Intelligence

To establish trust with end users, following principles are essential to follow: These principles include:^6^ **(i) Transparency**: AI systems and their decision-making processes should be clear and comprehensible to patients, healthcare professionals, and regulatory bodies; **(ii) Beneficence and Non-malfeasance**: AI should be employed to enhance patient well-being while preventing any harm; **(iii) Justice and Fairness**: AI systems should be developed and implemented in a way that ensures impartiality and prevents discrimination; **(iv) Patient Autonomy and Consent**: Patients should have control over their healthcare choices, and their data should only be utilized with informed consent; an: **(v) Privacy and Confidentiality**: Patient information must be handled with complete security, ensuring strict privacy protection.

### Ethical challenges associated with artificial intelligence in healthcare

The ethical challenges linked to artificial intelligence (AI) in healthcare encompasses the apprehensions such as privacy, bias, discrimination, and the extent of human judgment involved. The implementation of AI technologies introduces potential risks, including inaccuracies and data breaches, which can have severe consequences for patients. Notably, lack of comprehensive regulations addressing the legal and ethical dimensions of AI's role in healthcare, underscoring the necessity for thorough examination of this critical issue.^7^

Ethical issues in AI data handling. Use of Electronic health records (EHRs) can enhance scientific research, healthcare quality, and clinical efficiency, they also pose risks such as potential hacking and misuse of data. Additional ethical concerns involve determining ownership of personal health records, deciding with whom and when this information is shared, and establishing the necessity for obtaining patient consent.^6^

Privacy and Data Protection. In November 2022, one of India’s leading hospitals, All-India Institute of Medical Sciences, New Delhi, had its computer servers knocked out by a ransomware attack that targeted its services like patient registration, online appointments, diagnostic report generation, billing, and administrative systems, such as salary disbursal and drug procurement etc. For approximately 2 weeks, these services managed manually. Even though online services have been resumed, with data restored from a backup server but the personal data of more than 30 million patients and healthcare workers may have been compromised. Globally numerous act and regulations are available to guide and protect the data. It includes the following: (i) General Data Protection Regulation: Applicable only in the European Union but can be used as a guide by other nations; (ii) Global Initiative on Ethics of Autonomous and Intelligent Systems: Aimed at formulation of a set of standards and principles for Autonomous and Intelligent Systems, to make them secure, ethical, and advantageous to society at large; (iii) Health Insurance Portability and Accountability Act: This act was passed as a federal law for formulating national standards for dealing with sensitive patients' health information and prohibiting its disclosure without the patient's consent or knowledge. 

Informed Consent and Autonomy must be given voluntarily, for a specific purpose, and without ambiguity. The growing use of AI in healthcare has further intensified concerns regarding this issue. In alignment with the principle of autonomy, patients must be fully informed to make independent and well-informed healthcare decisions.^13^

Social Gaps and Justice. The advancement of AI has imposed another challenge to societies, as difference of advancement in developing and developed countries, loss of jobs of individuals in various areas due to growing robots and automated machines like surgical robots etc.

Issues related to Consultation and understanding of health problems. Integration of artificial intelligence in healthcare appears challenging and impractical. The presence of uniquely human emotions makes it unlikely that humans and medical robots will develop together rapidly. Many a times, Health professionals do consultation in collaboration with their seniors and juniors to guide, learn or sometime for the benefit of the patients. This is difficult to think that the traditional human relations will be replaced by machine human interaction. Although the machine (Robot) will not have the feeling, kindness, compassion which are the quality of human being and help in healing process of the patient. Hence it is one of the major issue related to Artificial intelligence in health profession.^12^

Use of Artificial intelligence in drug development: Artificial intelligence has gained success in the drug discovery but this is required large amount of information which may not be possible in some cases where the limited information or low quality of data is available as it may affect the reliability and validity of the results. It also raises the issue related to fairness and biases.^8^

Human values and AI in healthcare. Software engineering, which underpins most AI applications, often overlooks human values.^18^ Neglecting human values can lead to numerous ethical concerns. According to Schwartz's Theory of Basic Human Values, ten core values have been identified and validated globally.^20^ Research has pinpointed security, benevolence, universalism, and self-direction as the four most commonly cited values in recent software engineering publications.^17^

### Human values and corresponding ethical principles

Security. Security encompasses the sense of safety and stability for individuals, their relationships, and the broader community, embodying the principle of doing no harm. (i) Non-maleficence. The principle of non-maleficence focuses on minimizing potential harm, including discrimination, privacy breaches, and physical harm.^16^ Current AI guidelines prioritize avoiding harm over doing good, highlighting the importance of preventing negative outcomes.^21^ However, the rapidly evolving nature of AI in healthcare raises concerns that potential harms may only be addressed after they occur. Safety is a top priority in healthcare AI, particularly given the limited empirical evidence supporting many initiatives.^22^ Technical issues, such as AI malfunctions or network failures, can lead to unintended harm. Moreover, AI's lack of cultural or interpersonal sensitivity may compromise the relationship and can cause emotional sufferings to individuals.^23^

Self-direction. Self-direction represents the idea of self-independence, encircling the ethical principles of freedom, dignity, and confidentiality, which enable individuals to make his / her independent choices. (i) Freedom and autonomy. Preserving autonomy and freedom means upholding individuals' rights to make informed decisions and revoke consent as needed,^24^ this includes transparently sharing relevant information and ensuring participants understand and agree to the terms.^25^ However, complex AI systems and vast datasets can make informed consent difficult to achieve.^26^ Furthermore, users of mobile health apps often assume their data is protected with the same rigor as traditional healthcare, but the fine print is often overlooked or not fully grasped;^27^ (ii) Dignity. Dignity involves upholding human rights and decency. This includes considering the well-being of developers who may be exposed to traumatic content without proper training.^28^ Additionally, dignity is relevant when individuals form bonds with AI, raising concerns such as patients mistakenly attributing human-like qualities to AI, experiencing unease due to robots' unsettling appearance, or struggling to terminate the therapeutic relationship safely;^23^ (iii) Privacy. Privacy is the right of every individual which requires careful protection and secure management of personal information. Sensitive health data demands confidentiality, a responsibility traditionally held by healthcare professionals.^28^ The advancement of AI in healthcare frequently clashes with the right to privacy, particularly regarding data collection, management, and utilization of social media information.^29^ Data acquisition can pose several ethical dilemmas. Data can be acquired from various sources (e.g., mobile device location services and online discussion board engagement),^24^ by machine learning algorithms,^22^ or via implicit methods (e.g., monitoring screen interactions or vocal pitch). A crucial issue revolves around individual comfort levels regarding data collection in these contexts, particularly when it occurs without their knowledge.^28^ Data management raises further concerns related to privacy which may be compromised due to careless security measures like unattended sensitive information in health setting, cyber-attacks, leaking of information through social media etc.^23^

Benevolence embodies the idea of promoting and preserving the well-being of oneself and others, rooted in core values such as doing good, accountability, reliability, openness, and unity; (i) Beneficence. The principle of beneficence is about advancing the welfare of individuals and society. While AI holds great promise for positive impact, concerns arise from its current limitations and potential influence on clinical judgment.^16^ AI's limitations need to be acknowledged. Clinicians, guided by their professional standards, can identify broader societal risks like domestic violence, child abuse etc. However, AI systems designed for specific tasks might overlook these critical indicators.^30^ AI-informed decisions may also have unintended effects on clinicians' judgments. For example, when radiologists are aware of a patient's high-risk genetic mutation, they tend to detect significantly more breast lesions on MRI scans.^31^ consequently; AI-driven predictions may influence clinicians' own risk assessments. If further screening or treatment is only offered to patients labeled high-risk by AI, without clinician oversight, it could create a self-perpetuating cycle;^32^ (ii) Responsibility and trust. Responsibility represents the accountability and liability, being transparent and acting with honesty to earn trust.^16^ when developing predictive models, it's crucial for creators to transparently outline their limitations.^30^ Notably, most suicide risk assessments can't accurately forecast when someone might attempt suicide, making it challenging to determine if intervention is required like involuntary restraint.^33^ The boundaries of accountability for AI systems are unclear, especially with complex "black box" algorithms that are difficult to interpret.^27^ This ambiguity raises questions about liability in cases where AI fails to detect a critical issue, such as a potential suicide - who bears the responsibility i.e. developer or the implementors? This issue remains unresolved.^33^ Other gray areas like discrepancies between AI-driven decisions and clinician judgments, such as when AI flags a patient as high-risk but the clinician disagrees.^33^ Unlike health professionals, AI systems lack inherent accountability and aren't capable of experiencing moral consequences, like emotional distress, resulting from poor decisions.^23^^,^^28^ and Trust can be compromised when AI produces numerous false positives or negatives; (iii) Transparency encompasses two key aspects: interpretability, which means to understand the decision-making process (the "how"), and explain ability, which refers to understanding the underlying reasons for the decision (the "why").^34^ The challenge lies in AI systems having limited interpretability and/or explains ability, as well as hidden shortcomings. While some AI algorithms, like regression models, offer more transparency whereas others, such as deep learning models built on vast datasets, often sacrifice interpretability for better performance.^24^ The problem with these "black box" algorithms is that they obscure the connection between inputs and outputs, making it difficult for humans to comprehend the decision-making process.^27^ The lack of transparency can have problems, such as patients being informed of a high risk of illness without understanding the underlying reasons. Some individuals are hesitant to receive such unexplainable high-risk assessments, as they can be distressing.^24^ Furthermore, if implementers cannot grasp the models, they may struggle to identify biases or challenge AI-driven decisions;^24^ (iv) Solidarity involves prioritizing the needs of people with low socioeconomic status and are in inaccessible community area which may require redistributing AI benefits to them.^16^ For example, NLP algorithms developed solely in English may exclude cultural groups that speak other languages, limiting their applicability.^25^ The use of AI in healthcare can have negative consequences for vulnerable populations. Some individuals, such as those with schizophrenia, may find AI-driven tracking and surveillance distressing.^27^

Universalism reflects a commitment to promoting human dignity and protecting the planet, guided by ethical principles that prioritize fairness, justice, and long-term sustainability. (i) Justice and fairness in AI development involve ensuring diverse representation in research, design, and development to prevent discrimination against vulnerable groups and protect the right of individual.^16^ If AI systems aren't developed with diverse populations in mind, they may overlook the requirements of diminished groups.^2^ Gaps in training data can arise from limited representation of non-binary individuals in electronic health records or from marginalized communities with restricted access to healthcare.^33^ Moreover, training data may reflect systemic biases related to human characteristics which can exacerbate existing disparities if used for predictions.^26^^,^^33^^,^^34^ Economic factors, such as varying billing rates, can also impact data collection and perpetuate biases.^34^ Additionally, social media data may skew towards younger, Caucasian populations, potentially neglecting the health needs of other groups.^35^^,^^36^ A critical concern is whether implementers can challenge AI-driven decisions, particularly in high-risk situations like suicide prediction. Given these risks, relying solely on AI for decision-making has been criticized for lacking human oversight and accountability.^37^

Sustainability in AI development means taking into account the environmental impact and striving to minimize the environmental foot print of AI projects. Although AI is beneficial in predicting the disease outbreaks, investigations, patient health care etc but still its implementation requires careful consideration of privacy of data, unintended consequences and data biases etc. Also, without concrete examples of AI in healthcare, it's challenging to define what sustainable AI in this field would look like. ^16^

Implications of Artificial Intelligence in Health Care: 

The World Health Organization (WHO) is urging careful consideration when utilizing large language models (LLMs) powered by artificial intelligence (AI) to safeguard human well-being, safety, autonomy, and public health. It's crucial to assess the risks associated with relying on LLMs to expand access to health information, support decision-making, or boost diagnostic capabilities in resource-limited areas, ultimately protecting people's health and reducing disparities. Rushing to adopt unproven systems can result in mistakes by healthcare professionals, harm to patients, and erosion of trust in AI, which could undermine the long-term benefits of these technologies.^16^ To ensure safe, effective, and ethical use, rigorous oversight is necessary to address concerns surrounding these technologies: ^38^

- The training data for AI systems can contain inherent prejudices, leading to flawed or deceptive outputs that might compromise health, fairness, and social inclusivity.
- LLMs produce answers that seem convincing and trustworthy, but they can be entirely wrong or contain significant mistakes, particularly when it comes to health information.
- LLMs might rely on training data that wasn't authorized for this purpose, and they may not safeguard sensitive information, including personal health details, shared by users when interacting with an application.
- LLMs can be exploited to create and spread highly believable false information in various formats, including text, audio, and video, making it challenging for people to distinguish it from trustworthy health information.
- As WHO embraces innovative technologies like AI and digital health to advance human well-being, they stress that policymakers must prioritize patient safety and security as tech companies develop and market large language models.

WHO suggests that these issues need to be resolved and concrete benefits demonstrated before large language models are widely adopted in everyday healthcare and medicine, whether by individuals, healthcare professionals, or those managing health systems and policies.^16^ AI's influence on healthcare is distinctive, but the question remains whether its use will be morally sound. While AI has the potential to reform disease diagnosis, risk prediction, personalized treatment, remote monitoring, and automated triage, it also poses substantial risks to patient safety and the healthcare sector's trustworthiness. These ethical concerns stem from three main areas: (a) the limitations and biases in healthcare AI's underlying evidence (knowledge gaps); (b) AI's potential to redefine health, healthcare, and medical practice (value-based concerns); and (c) the opaque nature of AI development, which hinders accountability and transparency (accountability concerns).^17^

Some areas of implication of Artificial Intelligence in Nursing are as follows:

- Clinical Nursing- AI application in nursing is an opportunity as well as a challenge in the health care system. It helps in decision making, critical analysis, assessment, monitoring but it cannot replace a nurse’s own decision making and skills. Although it is a responsibility of nurses to make effective use of AI with transparency, effective communication with their patients and respect their autonomy.^39^
- Evidence based Nursing- Use of AI in clinical nursing equip the nurses with statistical data and suggestions based on available evidences as well as research studies. Based on information nurses can take prompt and accurate decision resulting in improved patient outcome.^40^
- Patient Satisfaction- AI technology has provided an extremely valued support system for the treatment of patients by reducing the cost of services, critical analysis, real time monitoring, work flow optimization, prompt action on any deviation, quick notification to health care provider and moreover it has enhanced the satisfaction level of patients as well as their family members.^41^^,^^42^
- Burden of care & Documentation- Although the use of AI like electronic health records facilitates the documentation burden and allows the more direct patient care, predictive analysis through AI, help in early intervention etc. Still there are challenges about the ethics, nurse patient relationship, privacy of data and dignity in relation to the acceptance to the AI.^43^ Hence clear guidelines and training of nurses are required for the integration of AI in the health care system in relation to the cultural and regulatory differences around the world.
- Robotics & Nursing- Artificial intelligence has been integrated with robotic processes which also increases the success of any critical procedure and decision making. Robotic implication with AI technology terminates the chances of human error and delayed decision-making process. ^44^
- Tele Nursing- Artificial intelligence through use of mobile application has been a significant contribution for the facility to provide various health care facility anywhere with low burden of infrastructure. It has also enhanced the cost effectiveness, efficiency and on spot analysis facility for the nurses.^45^
- Nursing Education- Whereas AI equipped nursing education tools, applications, simulation platforms, wearable technologies virtual reality-based platforms, chatbot systems has been found to be helpful for students to learn about patients’ conditions, critical analysis of their clinical changes, real life demonstration and continuous learning that makes them well skilled and knowledgeable to deal with real patients in clinical set up.^46^

**Conclusion.** Use of Artificial Intelligence in health settings can transform the care by improving accuracy in clinical diagnosis, treatment plans, and decision-making. However, ethical issues related to artificial intelligence must be addressed must be addressed. AI can augment clinical judgment, enabling healthcare professionals to make more informed decisions. In resource-constrained settings, AI can help with screening and evaluation, particularly when medical expertise is limited. Unlike human decision-making, AI-driven judgments are systematic and algorithm-based, ensuring accountability - not of the machine, but of its developers and users. While AI poses moral dilemmas, it's likely to complement or replace existing systems, ushering in a new era of healthcare. Not leveraging AI may be unscientific and unethical, given its potential benefits.

Abbreviation used: AI- Artificial Intelligence, LLM- Large Language Model, EMR- Electronic Medical Records, EHRs- Electronic Health Records.
